# A cost-effective and open-source near-field electrospinning system with a graphical user interface

**DOI:** 10.1016/j.ohx.2025.e00691

**Published:** 2025-09-11

**Authors:** Cristian Castillo-Velásquez, Carlos Fuhrhop, Mario E. Flores, Sebastian Brauchi

**Affiliations:** a Universidad Austral de Chile, Facultad de Medicina, Instituto de Fisiología, Laboratorio de Biofísica Celular, Edificio Ciencias Médicas, Campus Isla Teja, Valdivia, Región de Los Ríos, Chile; b Universidad Austral de Chile, Facultad de Ciencias de la Ingeniería, Instituto de Electricidad y Electrónica, Campus Miraflores, General Lagos, 2086 Valdivia, Región de Los Ríos, Chile; c Universidad Austral de Chile, Facultad de Ciencias, Instituto de Ciencias Químicas, Laboratorio de Polímeros, Campus Isla Teja, Av. Independencia 631, Valdivia, Región de Los Ríos, Chile

**Keywords:** Near-field electrospinning, Open-source hardware, Syringe pump, 3D printing, Graphical user interface, Electrospun fibers

## Abstract

Electrospinning is a versatile technique widely used in biomedicine and electronics. Here we describe the design and construction of a low-cost Near-Field Electrospinning System (NFES) using open-source technologies, including 3D printing and open-source hardware and software. The system features a modified 3D printer for precise needle and mobile collector control, along with an Arduino-driven syringe pump to regulate the flow of the polymeric solution. A custom user interface ensures optimal conditions during operation. Proof-of-concept tests demonstrate the system capability to fabricate and functionalize microfibers using a polyethylene oxide solution in distilled water.

## Graphical abstract

**Figure:**
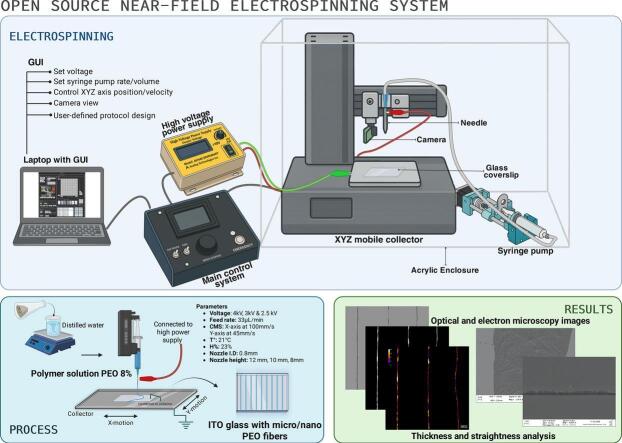

Specifications table

**Table:** 

Hardware name	*Low-cost near-field electrospinning machine*
Subject area	- Engineering and material science - Educational tools and open source alternatives to existing infrastructure
Hardware type	- Electrical engineering and computer science - Mechanical engineering and materials science
Closest commercial analog	NFES M01‑001 – Foshan Nanofiber Labs
Open source license	*CC BY‑SA 4.0*
Cost of hardware	*USD 1200*
Source file repository	*https://doi.org/10.17605/OSF.IO/GK6PC*
OSHWA certification UID *(OPTIONAL)*	

## 1 Hardware in context

Within the field of nanotechnology, electrospinning stands out as a versatile technique for producing micro- and nanometer-sized structures with a wide range of applications. First patented by John Cooley in 1900 [1], this method involves injecting a solution—typically a polymer dissolved in a suitable solvent—through a fine needle while applying a strong electric field between two electrodes. The electric field charges the liquid electrostatically, creating a thin jet once the electrostatic energy surpasses the surface tension [2]. As the jet elongates and dries on its way to the collector, it forms micro- or nanofibers with diverse sizes and morphologies. Notably, the jet emerges when the energy from the applied electrostatic charge exceeds the energy needed to maintain surface tension [2,3]. Compared to other material manufacturing methods, electrospinning offers several advantages, such as low cost and remarkable versatility. It enables the fabrication of nano- and microfibers from a wide array of materials that can be easily customized and further functionalized for specific applications.

In biomedicine, ultrafine fibers produced by electrospinning are widely employed as scaffolds for tissue engineering and drug delivery systems, supporting localized and sustained release of therapeutic agents. This versatility is highlighted by recent studies. For example, electrospun fibers have been used to promote angiogenesis through gene-activated scaffolds [4], to achieve biphasic anticancer delivery with polyblend systems [5], and to develop multifunctional wound dressings with shape memory and antibacterial properties [6]. Additionally, bilayer constructs integrating nanofibers loaded with antibacterial and anticancer drugs have been proposed for postoperative care of cutaneous carcinomas [7]. Reviews continue to emphasize advances in tailoring electrospun biopolymer-based biomaterials for enhanced biomedical performance [8]. Altogether, these examples underscore electrospinning as a highly adaptable technique for crafting fibrous platforms with tunable biological functions, solidifying its role in the development of next-generation therapeutic scaffolds.

Electrospun fibers have also been engineered into highly sensitive transducers. Among the most prominent are BioFETs, which couple micro- or nanofibers to field-effect transistors, and BioCPSs, which integrate such fibers with coplanar waveguides. These devices have been applied to detect glucose, plasma proteins, hormones, and even viral particles with outstanding selectivity [9,10]. Their exceptionally high surface-to-volume ratio, intrinsic porosity, and customizable surface chemistry maximize the density of recognition sites and accelerate mass transport to the active interface. This makes polymer-nanofiber biosensors both inexpensive and highly responsive. Sub-micromolar glucose detection has already been demonstrated [11]. Meanwhile, two complementary reviews map the broader landscape: Halicka et al. [12] catalogue assays for dopamine, streptavidin, vitamin D_3_, MUC1, nicotine, progesterone, and many other clinically relevant analytes, whereas Aliheidari et al. [13] highlight label-free architectures that exploit impedance, piezoresistive, and optical transduction. These approaches simplify fabrication and enable real-time monitoring in resource-limited settings.

However, a primary challenge of traditional electrospinning is achieving precise control over system parameters to produce fibers with specific sizes, diameter distributions, and morphologies. To address this, the near-field electrospinning (NFES) technique was introduced as an advanced variant, offering precise control over fiber deposition [14]. NFES has proven remarkably effective for producing highly aligned, uniform, and well-structured nanofibers [15]. Achieving such precision, however, requires careful optimization and control of multiple parameters, including the applied voltage, polymer concentration, solution viscosity, ambient temperature and humidity, the distance between the syringe tip and the collector, the type of collector, and its movement dynamics [16]. These factors collectively determine the morphology and properties of the resulting nanofibers, underscoring the need for precise parameter tuning in NFES.

While several commercial NFES systems are available for laboratory-scale applications, they are often prohibitively expensive, with prices ranging from USD 20,000 to 50,000. As a result, many laboratories choose to build custom systems using readily available components [[17], [18], [19]]. A basic NFES setup typically consists of a syringe pump, a high-voltage DC power source, a mobile platform with two or three axes of movement, an adjustable syringe needle holder, and a safety enclosure for high-voltage isolation. In contrast, open-source initiatives in far-field electrospinning have enabled a variety of low-cost platforms for producing nanofiber scaffolds. These include modular or 3D-printed systems, devices with interchangeable collectors, and setups adapted for biomedical and sensing applications [[20], [21], [22], [23], [24]]. Additionally, melt electrowriting setups represent the closest approach to NFES patterning, though they rely on molten polymer deposition without the characteristic electric-field-driven jet seen in NFES [25]. This context highlights the limited availability of open-source systems specifically designed for NFES, motivating the development of the platform presented here. Table 1 contrasts key performance metrics, environmental controls, and costs of representative commercial NFES platforms with this open-source alternative.

**Table 1:** Comparative features and capabilities of commercial NFES platforms versus the presented open-source system.

**System / Manufacturer**	**Main mode**	**XYZ travel (mm) & positional resolution**	**Fibre Ø range (µm)**	**Environment & vision add–ons**	**Software / GUI**	**Flow–rate control**	**Approx. price (USD)**
TL–03 Melt Electrospinning Direct–Writing Machine – Tong Li Tech	NF (melt)	150 × 150 × 100 / 0.1 µm	0.2 – 40	Closed cabinet, on–line microscope	Proprietary Windows GUI	Motorised micro–pump	96 000
AutoBio 2000 DIW (electrospinning option) – MakerPi	NF option + DIW	300 × 200 × 100 / ±10 µm	n/d (Ø ≈ > 1)	Temp–controlled chamber; 4–head camera	Touch GUI + slicer	Digital pressure (±0.2 kPa)	40 000 – 50 000
NFES M01–001 – Foshan Nanofiber Labs	NF (solution + melt)	200 × 200 × 100 / ±0.02 – 0.05 mm	0.5 – 200 (solution) 0.5 – 500 (melt)	Temp/–humidity to 50 °C, HD microscope	PC touch GUI	Syringe (0.1 mL h^−1^ min) / pneumatic feed	20 000
This work – open–source NFES	NF (solution)	200 × 200 × 180 / 50 µm (belt)	5 – 40 (PEO demo)	Acrylic hood, needle–tip webcam	Java GUI + 3.5″ HMI	Stepper syringe (≦ 0.03 mL min^−1^)	1 200

## 2 Hardware description

Despite the growing popularity of electrospinning, commercially available NFES systems and published open-source designs remain limited—particularly those featuring a mobile platform collector. Most existing setups rely on expensive components, such as commercial syringe pumps, motorized platforms with dedicated software, and specialized control systems. Consequently, researchers often face challenges in achieving precise coordination between platform movement, solution delivery, and voltage regulation—critical parameters for producing fibers with consistent morphologies. This complexity frequently hinders the reproducibility of experiments due to the manual synchronization required across multiple components.

To address these limitations, an open-source near-field electrospinning system was developed. The platform integrates precise flow-rate and volume control through a 3-D-printed syringe pump, regulates the high power supply voltage, and drives XYZ-axis motion with components adapted from an affordable desktop 3-D printer. Custom software with a graphical user interface (GUI) governs every module and offers real-time visualisation via an inexpensive webcam focused on the needle tip where the jet emerges. The GUI lets users create, coordinate, and store action sequences based on their own experimental protocols; these protocols can be saved and reloaded as needed, enabling highly precise and repeatable experiments without manual parameter adjustments.

The main advantages of the presented hardware system include:

● Simultaneous control of flow rate, volume, voltage, and platform movement, enhancing experimental repeatability.
● Reduced costs through the use of affordable components and fully open-source software and hardware.
● A user-friendly GUI that simplifies parameter adjustments, experiment coordination, and real-time visualization via webcam.
● Flexibility to develop and test novel electrospinning protocols and configurations.
● Potential for collaborative research and standardized protocol sharing within the scientific community.

An additional advantage of the system is its modular architecture. The 3-D-printed, slip-fit needle holder is supplied for a standard blunt-tip syringe, but the openly available STL file can be readily re-designed and re-printed to accept coaxial, triaxial, or other custom nozzles, broadening the platform to multi-fluid and core–shell electrospinning. All design files, firmware, and GUI source code are distributed under an open licence, enabling users to adapt the holder geometry, add supplementary syringe pumps, or integrate bespoke extrusion hardware without altering the core electronics. This versatility facilitates advanced protocols—such as coaxial core–shell spinning for controlled drug release [26] and triaxial gradient-fibre fabrication for mechanically graded implants [27]—while preserving the system’s low cost and ease of use. Moreover, tailored adaptations, such as the implementation of sterile working conditions, can be easily achieved through standard laboratory practices (e.g., UV sterilization, sterile tubing, and disposable needles), which remain external to the core NFES design and can be incorporated according to specific user needs.

**Fig. 1:**
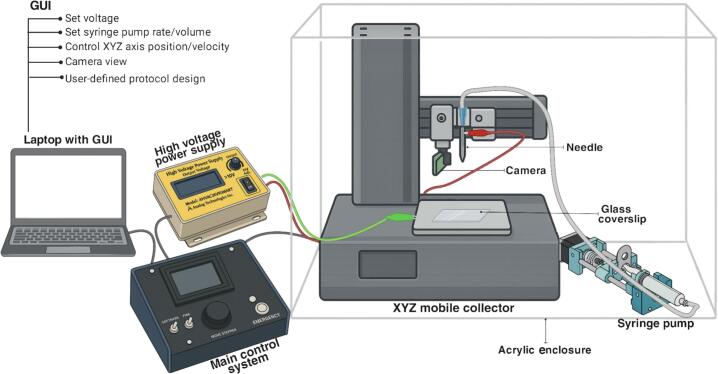
General schematic of the NFES system.

Fig. 1 provides a schematic overview of the system architecture, which includes six integrated modules: a high-voltage power supply, a syringe pump, a modified 3D printer serving as the XYZ mobile collector, a main control system, an acrylic enclosure, and the GUI. Specifications for each module and the overall system are detailed in Table 2.

**Table 2:** Specifications of the NFES system.

**Parameter**	**Value**
Voltage range	0–30 kV
Collector type	Flat XYZ platform
Collector velocity	200 mm/s max (X axis)
Syringe pump velocity	45.98 mL/min (20 mL syringe)
Control type	User panel (Touch screen and buttons); GUI software.
Safety	Transparent acrylic box
Price (USD)	1200

### 2.1 High voltage power supply

**Fig. 2:**
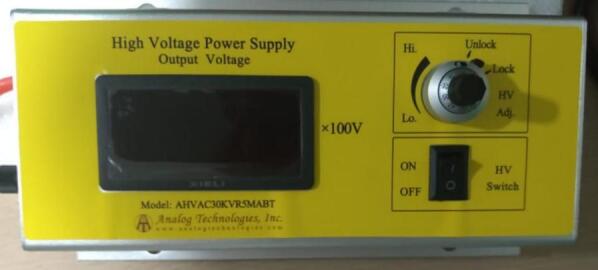
High voltage power supply AHVAC30KVR5MABT (Analog Technologies inc).

For the electrospinning process, a high-voltage power supply (HVPS) from Analog Technologies Inc. (model AHVAC30KVR5MABT, Fig. 2) was employed. This unit provides a maximum output voltage of 30 kV. The voltage is adjustable via a multi-turn potentiometer located on the front panel. In addition, the system includes a built-in digital display that functions as a voltmeter, providing real-time output voltage readings. Specifications are outlined in Table 3.

**Table 3:** HVPS specifications.

**Parameter**	**Value**	**Unit**
AC input power supply voltage	90–23	V
Output voltage range	0–30	kV
Output current	0–10	mA
Full Load Efficiency	>70 %	
Operating temperature range	−20 to 55	°C
External Dimensions	210x120x50	mm
Weight	1192	g
Price	559	USD

The HVPS was modified to enable digital reading and control of the output voltage via the main control system. This was accomplished by connecting the HVPS’s internal control ports directly to the control system, bypassing the factory-installed multi-turn potentiometer and voltmeter. A new port was added to the front of the HVPS enclosure to facilitate connection through a 4-wire cable. By incorporating analog-to-digital (ADC) and digital-to-analog (DAC) converter modules within the main control system, precise monitoring and adjustment of the output voltage was achieved. Furthermore, the modification allows for easy reversion to the original factory configuration by inserting a jumper into the newly added port.

### 2.2 Syringe pump

**Fig. 3:**
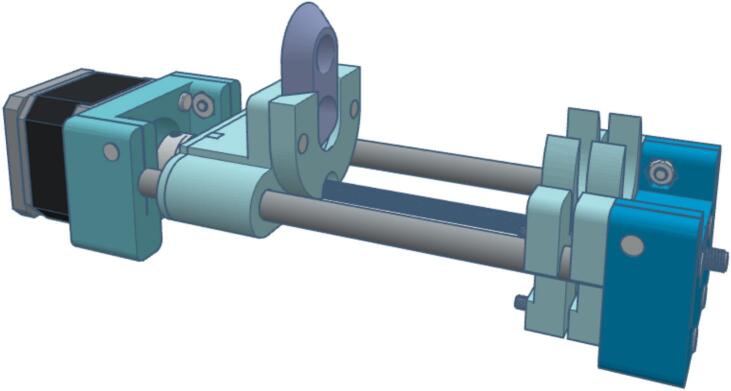
Syringe pump design.

The syringe pump is powered by a NEMA 17 stepper motor with a 1.8° step angle, providing precise linear actuation to displace the syringe plunger with controlled force. The mechanism is driven by a rotary shaft coupled to an M5 lead screw, which actuates a rail mounted on two 8 mm × 150 mm stainless steel rods. This rail securely holds the syringe plunger flange and applies horizontal force during operation. The pump design was modeled in Tinkercad and fabricated from 3D-printed components. It is a customized version of Bas Wijnen’s open-source system [28], which has been applied in neuroscience [29] and microfluidic research [30]. Modifications include the integration of an endstop sensor and adjustments to accommodate 8 mm rods and an M5 lead screw, improving overall stability and mechanical performance. Fig. 3 illustrates the design.

The motor and endstop sensor cables are connected to a GX16 connector, which links to the main control system. The motor is driven by a DVR8825 stepper driver, while the endstop sensor is wired to a digital input on the microcontroller.

Some crucial parameters are described as follows:

● The linear displacement per pulse L_D_[*mm/pulse*] of the syringe pump is given by Eq. (1), which depends on the thread pitch of the lead screw LS_P_[*mm/rev*], the motor step size SM_size_[*step*], and the microstepping mode μS[*step/pulse*] configured on the stepper controller.

(1) $$L_{D}\left[\mathrm{mm}/pulse\right]=\frac{LS_{p}.\mu S}{SM_{\mathrm{size}}}$$

● The flow rate FR[*mL/*min] is given for Eq. (2), which depends on the stepper motor speed SM_speed_[*RPM*], the syringe inner diameter S_D_[*mm*], and the aforementioned parameters:

(2) $$FR\left[\mathrm{mL}/min\right]=\pi \cdot {\left(\frac{S_{D}}{2}\right)}^{2}\cdot LS_{p}\cdot SM_{\mathrm{speed}}\cdot 0.001$$

● Using Eq. (2), Eq. (3) can be derived to calculate the stepper motor speed necessary to achieve a desired flow rate:

(3) $$SM_{\mathrm{speed}}\left[\mathrm{RPM}\right]=\frac{1000\cdot FR}{\pi \cdot {\left(\frac{\mathrm{sp}}{2}\right)}^{2}\cdot LS_{p}}$$

Table 4 displays the specifications of the syringe pump and the maximum and minimum flow rates it supports.

**Table 4:** Syringe pump specifications.

**Parameter**	**Symbol**	**Test condition**	**Value**	**Unit**
Linear displacement	L_D_	LS_P_= 0.8 mm (M5)	0.001	mm/pulse
Stepper motor speed	SM_speed_	NEMA 17 1.8° stepper motor with DVR8825, 1/4 μSmode.	max: 200	RPM
Flow rate	FR	S_D_: 14.5 mm (10 mL syringe).	min: 1.32	μL/min
			max: 26.41	mL/min
		S_D_: 19.13 mm (20 mL syringe).	min: 2.3	μL/min
			max: 45.98	mL/min
		S_D_: 26.7 m (60 mL syringe).	min: 4.48	μL/min
			max:89.58	mL/min

### 2.3 XYZ mobile collector

For the mobile collector, the mechanical infrastructure of an affordable desktop 3D printer was repurposed, rather than constructing a custom CNC system from scratch. This approach offered a more cost-effective solution while meeting all functional requirements. The design and development of a custom XYZ CNC system was estimated to cost approximately USD 600, whereas the selected M200 FDM printer model, priced at USD 200, provided sufficient mechanical capabilities. Although the printer’s original microcontroller was not used for operating the NFES system, its mechanical components proved adequate for precise platform movement. Detailed specifications of the XYZ mobile stage are provided in Table 5.

**Table 5:** XYZ mobile stage specifications.

**Parameter**	**Symbol**	**Test condition**	**Value**	**Unit**
X-axis linear displacement	L_X_	NEMA 17 1.8° stepper motor with DVR8825, 1/4 μS mode. 1 pulse.	min: 100	μm
Y-axis linear displacement	L_Y_		min: 10	μm
Z-axis linear displacement	L_Z_		min: 1	μm
X-axis velocity	V_X_	NEMA 17 1.8° stepper motor with DVR8825, 1/4 μS mode.	max: 200	mm/s
Y-axis velocity	V_Y_	min: 0.1 RPM	max: 50	mm/s
Z-axis velocity	V_Z_	max: 200 RPM	max: 5	mm/stie

Several modifications were made to the 3D printer, including the removal of the original microcontroller and the reconfiguration of its motors and endstop sensors. These components were rerouted to three GX16 connection ports—one for each motor and its corresponding endstop sensor—allowing external access for integration with the main control system. In addition, the printer’s original hot end was replaced with a custom 3D-printed component designed to hold the syringe needle, the connected electrode, and a webcam salvaged from an old laptop. The webcam is positioned near the needle tip to capture the formation of the Taylor cone during the electrospinning process. Its compact board design enables precise placement at an optimal focal distance, providing a low-cost and effective solution for real-time visualization of the jet.

### 2.4 Main control system

**Fig. 4:**
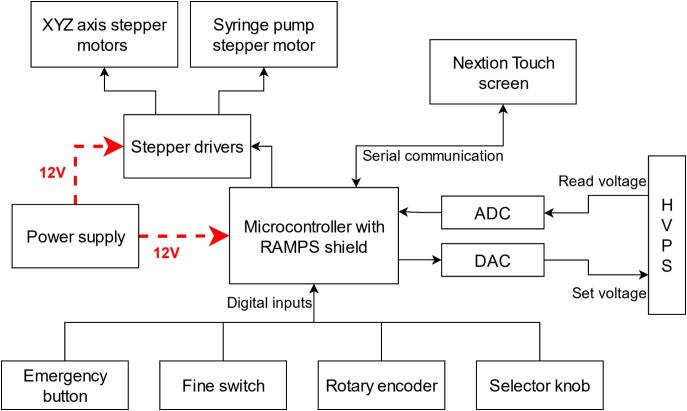
General diagram of the main control system.

The main control system consists of an Arduino MEGA 2560 microcontroller paired with a RAMPS 1.4 shield. The RAMPS (RepRap Arduino Mega Pololu Shield) board is specifically designed for 3D printing applications and offers robust motor control capabilities. In the present configuration, the RAMPS shield manages up to four stepper drivers: three dedicated to controlling the stepper motors for the X, Y, and Z axes of the mobile platform, and one for the syringe pump motor. In addition, the shield provides accessible digital inputs and outputs for peripheral devices such as a touchscreen, rotary encoder knob, selector knob, switch, and emergency stop button. A general schematic of the main control system and its connected peripherals is shown in Fig. 4.

The rotary encoder is used to adjust the parameter selected by the selector knob, allowing control of the motors corresponding to the X, Y, and Z axes, as well as the syringe pump (P). It also enables manual voltage adjustment of the power supply, which is activated by pressing the rotary encoder. A fine switch located on the left modifies the step size for parameter adjustment. When activated (in the upward position), it facilitates smoother and more precise tuning during encoder rotation.

These peripherals provide several advantages during experimental procedures, as they allow system adjustments to be performed without opening the safety enclosure. For example, the syringe pump can be manually operated to dispense or retract a specific volume of polymer solution until a small drop is formed at the needle tip—a necessary condition to initiate Taylor cone formation. The user may also conduct preliminary tests to determine the optimal Z-axis height, either to prevent collisions with the collector’s XY base or to set the needle at the proper distance for stable jet initiation. In the event of a collision with a sample object, the endpoint can be manually corrected using the XYZ controls.

**Fig. 5:**
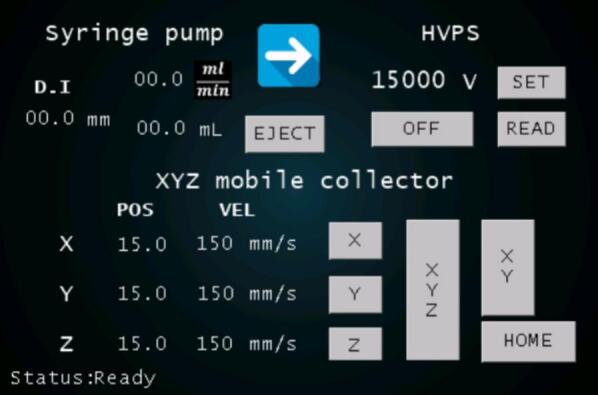
Touch screen main menu.

A 3.5-inch Nextion touchscreen was integrated for system control and monitoring of the electrospinning process. This interface enables users to operate the syringe pump, regulate and monitor the HVPS output voltage, and manage the movement of the XYZ mobile platform—facilitating fine adjustments without requiring external computer software. Fig. 5 displays the main menu of the touchscreen interface.

### 2.5 Acrylic enclosure

**Fig. 6:**
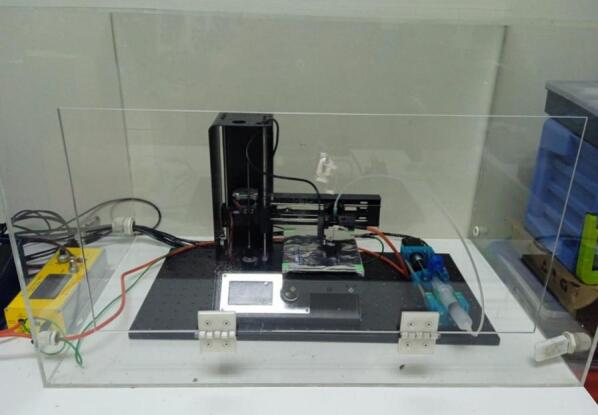
Photo of the acrylic enclosure.

An acrylic safety enclosure was designed to house the XYZ mobile collector, as illustrated in Fig. 6. The structure is assembled from six acrylic panels, one of which includes a hinged lid to provide user access to the system. The enclosure is equipped with two cable gland ports: one designated for the collector’s outgoing cables (including those for the motors and camera), and another for the incoming high-voltage electrode connection from the HVPS. While this configuration ensures both operator safety and convenient access for system setup and maintenance, the position of the door and cable glands may be adjusted according to user needs.

## 3 Design files summary

### 3.1 Electrical components

**Fig. 7:**
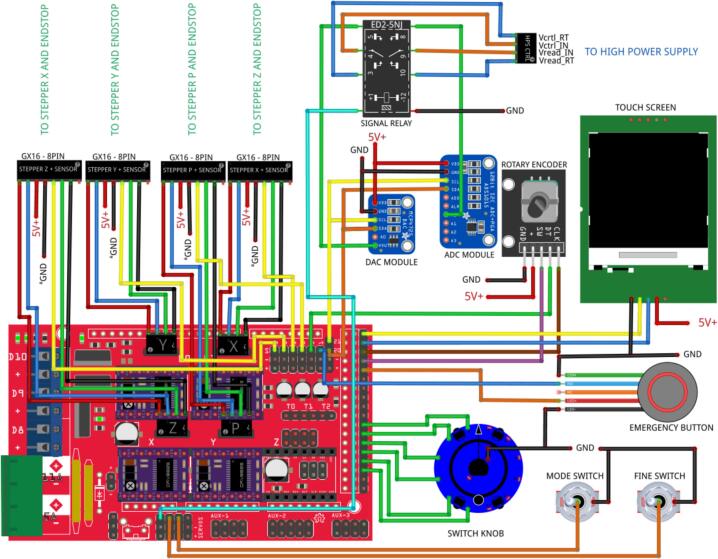
Main control unit schematic circuit.

Fig. 7 presents the schematic diagram of the electrical circuit corresponding to the main control unit. The electrical modifications applied to the high-voltage power supply (HVPS) are described in detail in the “Build Instructions” section.

### 3.2 Arduino firmware

**Fig. 8:**
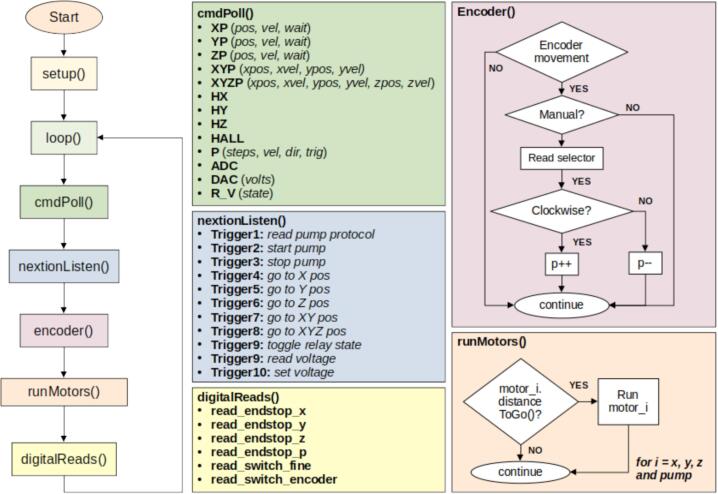
Arduino firmware flow chart.

Fig. 8 illustrates the algorithm flow of the Arduino Mega program. The.ino files, available for download from the OSF repository, are organized by functional modules and include detailed comments to enhance clarity. At the beginning of the main program, key parameters and variables are clearly indicated to facilitate user customization and system calibration.

### 3.3 Touchscreen firmware

The.HMI file for the Nextion touchscreen interface is available for download from the project's OSF repository. To upload the file to the screen, it must be copied onto a microSD card and inserted into the slot located on the back of the touchscreen. Upon powering the device, the screen will display a message indicating the upload progress. Once the upload is complete, the microSD card should be removed, the screen powered off, and then restarted to finalize the update.

### 3.4 Controller software with graphical user interface

**Fig. 9:**
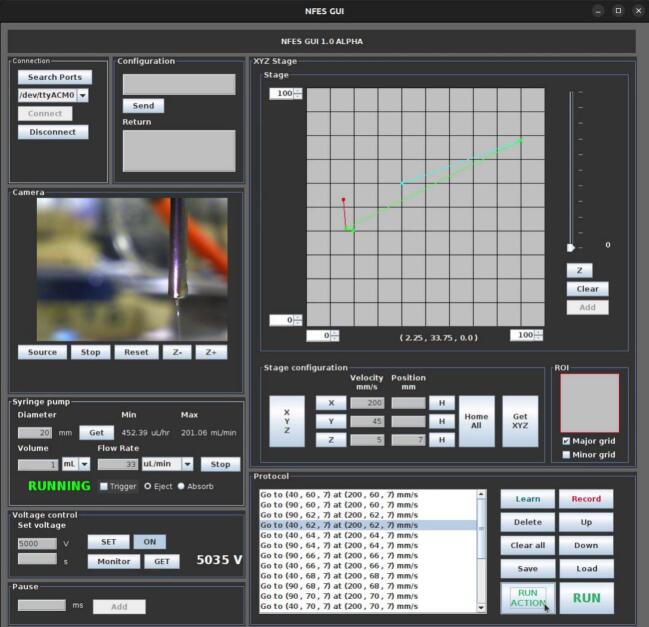
Screenshot of the graphical user interface.

A Java-based software application with a graphical user interface (GUI) has been developed to control the electrospinning process. This software communicates with the microcontroller via a serial port to transmit commands and includes a live streaming feature for the webcam focused on the needle tip. Fig. 9 shows the main interface of the GUI, which enables users to adjust parameters such as voltage, flow rate, and the position of the XYZ platform. Its key features include:

1. **Connection:** Automatically detects and lists available serial ports, allowing users to select and establish communication with the microcontroller.
2. **Configuration:** Supports manual command transmission for debugging and system configuration purposes.
3. **Camera control:** Offers settings for configuring the webcam stream, including cropping a region of interest (ROI) and zoom functionality.
4. **Syringe pump control:** Enables execution of syringe pump protocols based on user-defined parameters such as syringe inner diameter, volume, flow rate, and direction (eject or absorb).
5. **Voltage control:** Facilitates real-time adjustment and monitoring of the High Voltage Power Supply (HVPS), with an on/off safety button to prevent errors or allow emergency shutdowns.
6. **XYZ stage control:** Displays and manages needle position and trajectory with a grid-based interface for precise positioning, ROI adjustments, and velocity settings.
7. **Protocol management:** Tracks and organizes commands during experimental sessions with the following tools:
  a. **Learn mode**: Lists selected commands without executing them.
  b. **Record mode**: Lists all executed commands.
  c. **Delete**: Removes a selected item from the command list.
  d. **Up/Down:** Adjusts the order of items in the command list.
  e. **Clear All:** Clears the entire command list.
  f. **Save/Load:** Saves command lists to files or reloads previously saved lists.
  g. **Run Action/Run:** Executes selected or all commands from the command list.
8. **Pause:** Adds a pause command in “Learn mode” for command coordination.

### 3.5 Case of use

Once all peripherals—the main control board and the USB needle‑tip camera—are linked to the host computer, the user launches the Java GUI and selects the correct serial port. Pressing Connect unlocks the motion commands, which are verified with Home All: the X, Y and Z axes return to (0, 0, 0). In the Camera panel the video source is chosen, Live streaming is enabled, and Crop may be applied to centre the needle tip precisely in the preview window.

In the Syringe Pump module the operator enters the syringe’s inner diameter (mm) and clicks Get; the GUI immediately calculates the admissible speed limits. The total dispense volume and the flow‑rate—selectable in µL min^−1^, µL h^−1^, mL h^−1^, or mL min^−1^—are then defined and tested with Dispense/Det, which toggles between extrusion and retraction.

The High‑Voltage panel is initialised at 0  kV (Set), after which the desired operating potential (e.g., 4  kV) is programmed and activated with ON; Get polls the realtime output for safety monitoring.

Preparation of the deposition area begins in Stage Configuration by raising Z ≈ 30  mm (5  mm s^−1^), placing the substrate (glass slide or Al foil) on the platform, and positioning the needle at the centre using the velocity/position controls or by clicking the interactive plot. With Learn mode engaged the user marks the print‑field vertices and records the intended tool‑path (e.g., ten 5  mm parallel lines spaced 0.20  mm at the required stage speed). A typical run proceeds as follows: (i) move the needle to a peripheral start point; (ii) start the syringe pump to form a pendant drop; (iii) enable the high voltage and allow the jet to stabilise; and (iv) execute the stored sequence. The GUI automatically synchronises flow rate, stage motion and HV output, resulting in reproducible direct writing of aligned fibres over the defined workspace.

### 3.6 Mechanical parts

The syringe pump and needle support components were designed using TinkerCAD, a free browser-based 3D modeling platform developed by Autodesk. These components were fabricated using a Creality Ender 3 3D printer with 1.75 mm PLA filament. The printing parameters included a 0.4 mm nozzle diameter, a print temperature of 200 °C, a bed temperature of 50 °C, a layer height of 0.2 mm, and a 50 % infill density. The STL design files for the printed parts are summarized in Table 6 and are available for download from the project’s OSF repository.

**Table 6:** Design files of the syringe pump (SP) and the needle support (NS).

**Design file name**	**File type**	**Open-source license**	**Location of the file**
SP motor mount	STL	CC BY–SA 4.0	https://doi.org/10.17605/OSF.IO/GK6PC
SP bearing mount	STL	CC BY–SA 4.0	https://doi.org/10.17605/OSF.IO/GK6PC
SP carriage	STL	CC BY–SA 4.0	https://doi.org/10.17605/OSF.IO/GK6PC
SP carriage closer	STL	CC BY–SA 4.0	https://doi.org/10.17605/OSF.IO/GK6PC
SP body holder A	STL	CC BY–SA 4.0	https://doi.org/10.17605/OSF.IO/GK6PC
SP body holder B	STL	CC BY–SA 4.0	https://doi.org/10.17605/OSF.IO/GK6PC
SP plunger closer	STL	CC BY–SA 4.0	https://doi.org/10.17605/OSF.IO/GK6PC
SP plunger holder tab	STL	CC BY–SA 4.0	https://doi.org/10.17605/OSF.IO/GK6PC
NS carriage	STL	CC BY–SA 4.0	https://doi.org/10.17605/OSF.IO/GK6PC
NS camera holder	STL	CC BY–SA 4.0	https://doi.org/10.17605/OSF.IO/GK6PC
Screen frame	STL	CC BY–SA 4.0	https://doi.org/10.17605/OSF.IO/GK6PC

The description of the 3D printing parts is listed below:

● **SP motor mount:** Secures the stepper motor and one end of the two stainless steel axes.
● **SP bearing mount:** Holds the lead screw bearing and anchors the opposite end of the stainless steel axes.
● **SP carriage:** Secures the syringe plunger flange and accommodates the endstop sensor, the M5 nut, and the linear bearings.
● **SP carriage closer:** Seals the slots where the linear bearings are installed.
● **SP body holder A:** Supports the syringe barrel flange.
● **SP body holder B:** Anchors the syringe barrel flange and includes a stopper for the endstop sensor.
● **SP plunger closer:** Attached to the carriage to connect the plunger flange.
● **SP plunger holder tab:** Locks the plunger flange onto the SP plunger closer.
● **NS carriage:** Supports the needle and NS camera holder.
● **NS camera holder:** attaches the web camera to the NS carriage.
● **Screen frame:** Provides support for the touchscreen, allowing it to be mounted on the main control unit enclosure.

## 4 Bill of materials summary

The bill of materials is presented in Table 7, organized by modules and including the 3D-printed parts. A detailed bill of materials is provided in the supplementary materials and is also accessible through the project’s OSF repository.

**Table 7:** Bill of materials.

**Designator**	**Provider**	**Qty**	**Cost per unity (USD)**	**Total cost (USD)**	**Material type**
**XYZ mobile collector**					
Malyan M200 FDM Mini 3D printer	Amazon	1	199	199	Mechanical/Electronic
Notebook web camera	Recycled	1	5	5	Electronic
*Needle support “carriage”*	3D printed	1	1.19	1.19	PLA polymer
*Needle support “camera holder”*	3D printed	1	1.19	1.19	PLA polymer
**Main control unit**					
Arduino Mega 2560	Amazon	1	48.9	48.9	Electronic
RAMPS 1,4	Amazon	1	9.39	9.39	Electronic
DVR8825	Amazon	4	2.89	11.59	Electronic
1P6T Rotary Switch Selector	Amazon	1	8.49	8.49	Electronic
Toggle Switch SPST	Amazon	1	4.96	4.96	Electronic
Push Button Switch LED momentary latching	Aliexpress	1	2.57	2.57	Electronic
Nextion NX4832T035 3.5 in. HMI	Aliexpress	1	37.78	37.78	Electronic
12 V 5A Switching Power Supply	Aliexpress	1	4.57	4.57	Electronic
Ky-040 rotary encoder module	Aliexpress	1	1.13	1.13	Electronic
C14 panel-mounted plug AC electrical inlet module	Aliexpress	1	1.55	1.55	Electronic
ADS1015 ADC module	Aliexpress	1	1.23	1.23	Electronic
MCP4725 DAC module	Aliexpress	1	1.37	1.37	Electronic
Perfboard 8x12cm	Aliexpress	1	1.08	1.08	Electronic
Relay A5W-K	Aliexpress	1	0.96	0.96	Electronic
GX16 8pin Pin Male plug & Female socket	Aliexpress	9	0.42	3.78	Electronic
UL2464 Sheathed Wire Cable 6 cores 10 m AWG22	Aliexpress	1	17.45	17.45	Electronic
4 cores wire AWG22 5 m	Aliexpress	1	4.4	4.4	Electronic
USB type B panel mount	Aliexpress	1	4.97	4.97	Electronic
Sloped enclosure 200X90X165MM	Aliexpress	1	18.72	18.72	Enclosure
Cable USB type B 1,5m	Aliexpress	1	3.52	3.52	Electronic
*Screen frame*	3D printed	1	2.61	2.61	PLA polymer
**Syringe pump**					
LM8UU linear bearings	Aliexpress	2	0.75	1.51	Mechanical
625ZZ bearings	Aliexpress	2	0.21	0.42	Mechanical
M5 metric thread rod 150 mm	Aliexpress	1	1.4	1.4	Mechanical
Shaft coupler 5mmx5mm	Aliexpress	1	1	1	Mechanical
Limit Switch 2P with handle	Aliexpress	1	0.14	0.14	Mechanical
Needle 23G	Amazon	1	0.1	0,01	Stainless steel
Tubing polyethtlene 80,430 (0.085′'X, 0.128′' X, 0.0215)	A-M System	1	38	38	Polyethylene
*Syringe pump “motor mount”*	3D printed	1	10.18	10.18	PLA polymer
*Syringe pump “bearing mount”*	3D printed	1	5.2	5.2	PLA polymer
*Syringe pump “carriage”*	3D printed	1	4.01	4.01	PLA polymer
*Syringe pump “carriage closer”*	3D printed	1	1	1	
*Syringe pump “body holder A”*	3D printed	1	1.61	1.61	PLA polymer
*Syringe pump “body holder B”*	3D printed	1	1,67	1.67	PLA polymer
*Syringe pump “plunger closer”*	3D printed	1	1	1	PLA polymer
*Syringe pump “plunger holder tab”*	3D printed	1	1	1	PLA polymer
**HVPS**					
AC-DC HVPS 30 kV 5 mA AHVAC30KVR5MABT	Analog Instruments	1	559	559	Equipment
**Enclosure**					
Acrylic enclosure 80 cm x 60 cm x 50 cm (WLH) 5 mm	Leufulab	1	142	142	Acrylic
20 mm PVC cable gland	Aliexpress	2	0.5	1	PVC
**Total**				**1167.54**	

## 5 Build instructions

### 5.1 Syringe pump assembly

**Fig. 10:**
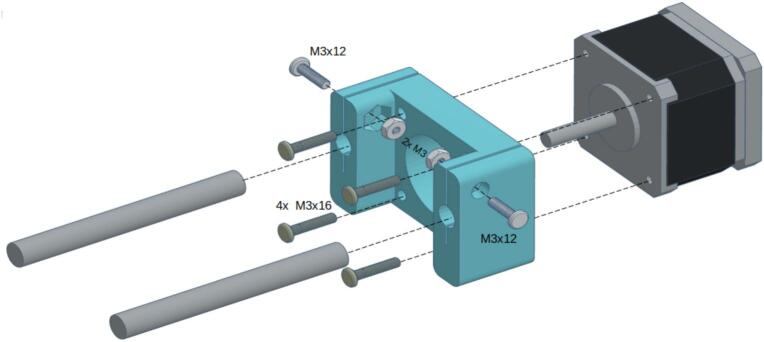
Motor mount assembly.

To construct the syringe pump, start by attaching the stepper motor to the 3D-printed component labeled “Motor Mount,” as shown in Fig. 10. Secure the motor using four M3 screws. Then, secure the 8 mm x 150 mm rods to the sides of the motor mount with two M3 screws and nuts on each side.

**Fig. 11:**
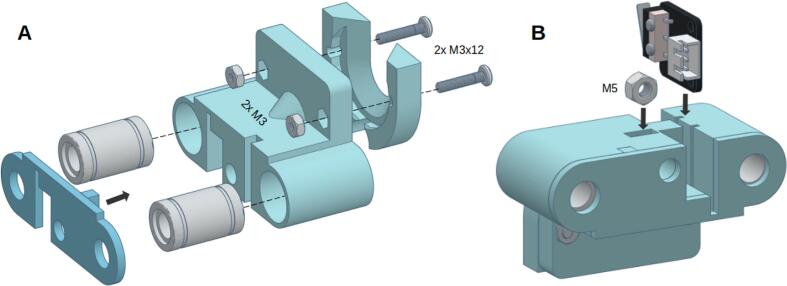
Carriage assembly.

The plunger carriage assembly comprises three 3D-printed components: the “Carriage,” “Carriage Closer,” and “Plunger Closer,” as illustrated in Fig. 11.A. Begin by inserting the two linear bearings into the “Carriage” and securing them with the “Carriage Closer”; if desired, you can use super glue for additional stability. Next, fix the “Plunger Closer” to the assembly using two M3 screws and nuts.

Finally, insert the M5 nut and the endstop sensor into their designated slots located on the lower part of the “Carriage,” as depicted in Fig. 11.B. Ensure all components are properly aligned and secured.

**Fig. 12:**
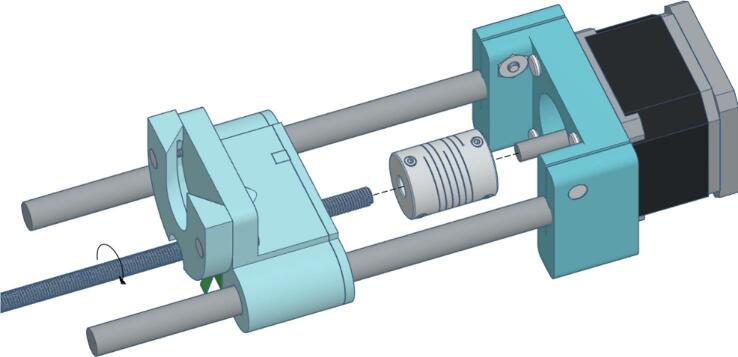
Carriage mounting.

Thread the M5 lead screw into the M5 nut on the carriage, then mount the carriage onto the guide rods. Secure the lead screw to the motor shaft using a shaft coupler, as illustrated in Fig. 12.

**Fig. 13:**
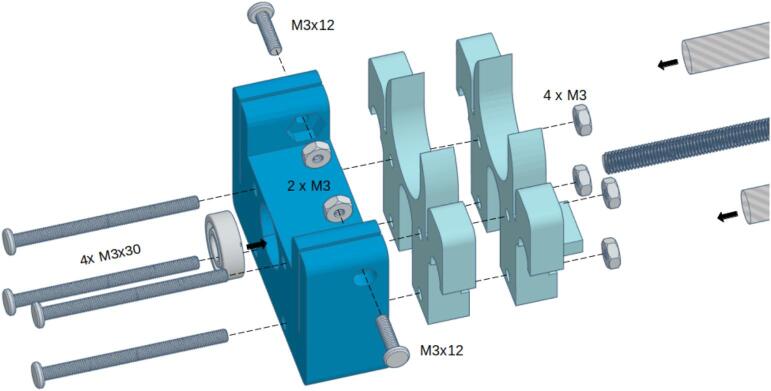
Bearing mount assembly.

The “Bearing Mount” 3D-printed component houses the linear actuator of the syringe pump. Begin by placing the bearing into its designated slot within the “Bearing Mount” and securing it with super glue. Next, insert the guide rods and the threaded lead screw into their respective slots on the mount. Use two M3 screws and nuts to secure the guide rods firmly in place. Then, position the “Body Holder A” and “Body Holder B” components onto the guide rods and secure them to the “Bearing Mount” using four M3 screws and nuts. Refer to Fig. 13 for a detailed view of the assembly.

**Fig. 14:**
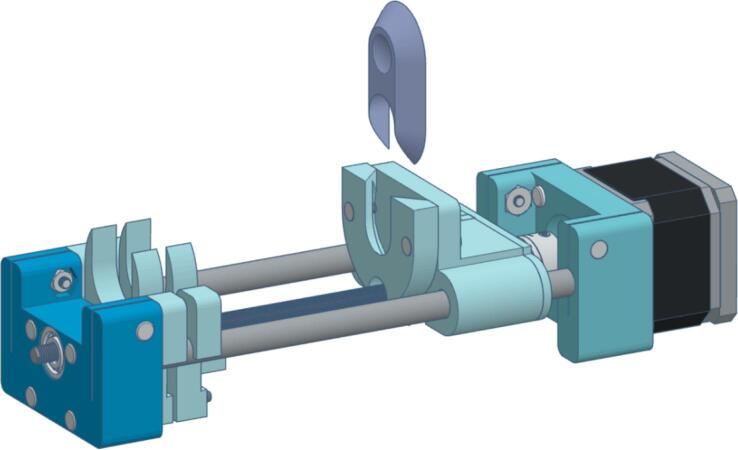
Syringe pump complete.

**Fig. 15:**
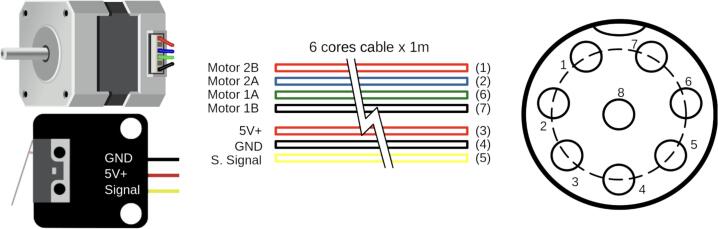
Syringe pump cable assembly.

The syringe pump should seem as shown in Fig. 14:

For motor and endstop sensor connection, use a 1-meter 6-core cable with a GX16-8 pin connector. Fig. 15 specifics wiring details.

### 5.2 HVPS hack

**Fig. 16:**
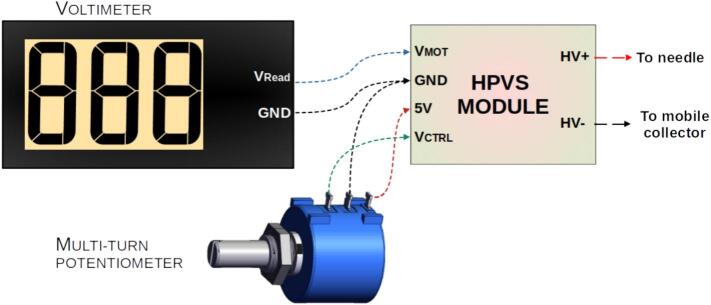
HPVS internal circuit diagram.

The internal circuitry of the HVPS was modified to enable both reading and controlling its output voltage, as shown in Fig. 16. Originally, a multi-turn potentiometer functioned as a voltage divider, adjusting the 0–5 V control signal feeding into the “Voltage Control Port” to regulate the 0–30 kV high voltage output (with a 1 mV/6V scale). In addition, a voltmeter module connected to the “Voltage Monitor Port” (Vmot) provided a 0–3 V output, corresponding to the 0–30 kV high voltage range (with a 1 mV/1V scale).

**Fig. 17:**
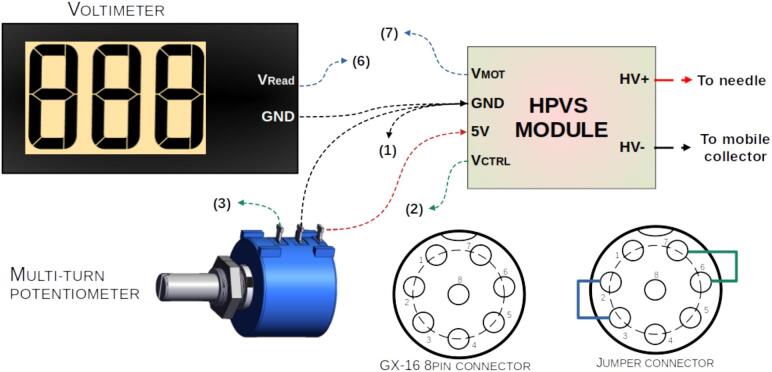
HPVS internal circuit diagram modification.

A GX-16 connector was added to facilitate access to the voltage control (VCTRL) and monitoring (VMOT) ports, enabling integration with a Digital-to-Analog Converter (DAC) module for voltage regulation and an Analog-to-Digital Converter (ADC) module for voltage monitoring of the HVPS. To ensure flexibility, a method to revert to the original configuration was also implemented. This involved wiring the voltmeter input and potentiometer output to the GX connector, allowing users to restore the original setup by simply connecting a jumper to the GX connector. The complete modification process is detailed in Fig. 17.

### 5.3 *XYZ mobile collector* modification

**Fig. 18:**
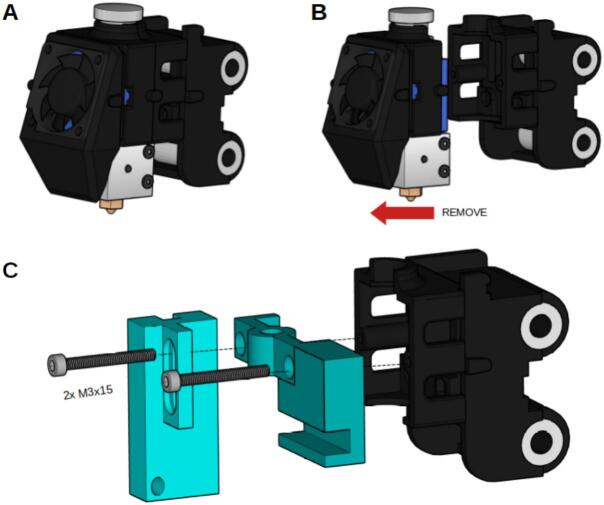
Needle and camera support assembly. A) The original 3D printer carriage. B. Detaching the hotend and fan. C) Attaching the Needle support and the Camera support.

To assemble the carriage, start by removing the hotend and fan from the 3D printer's carriage. Next, secure the “Needle Support” and “Camera Support” components using two M3 screws. Adjust the height of the camera support to ensure that the camera is properly focused on the needle tip, as illustrated in Fig. 18.

**Fig. 19:**
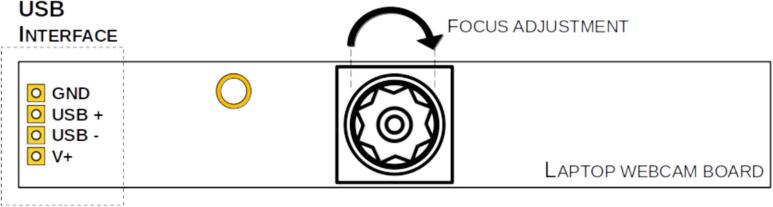
Web camera board adjustment and connection.

To image the electrospinning process, a laptop webcam module was selected as a cost-effective solution. The USB connection of the camera can be easily configured using the labeled pinout available on most boards, as shown in Fig. 19. Depending on the specific camera module used, modifications to the camera mount may be required to ensure proper fit.

Laptop webcams typically feature a fixed focal length intended for short distances, such as the gap between a user's face and the laptop screen. In the present setup, where the camera must be placed in close proximity to the needle tip, the default focal length is insufficient. However, this limitation can be addressed by adjusting the lens position—either by screwing it inward or outward—to achieve proper focus.

**Fig. 20:**
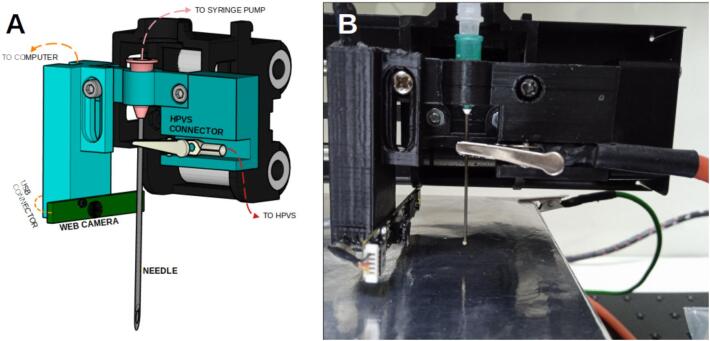
Needle and camera support assembled. A. Scheme of the final carriage. B) Photo of the carriage.

Secure the web camera board to the camera support using an M3 screw and nut. The entire carriage should be seen as shown in Fig. 20.

**Fig. 21:**
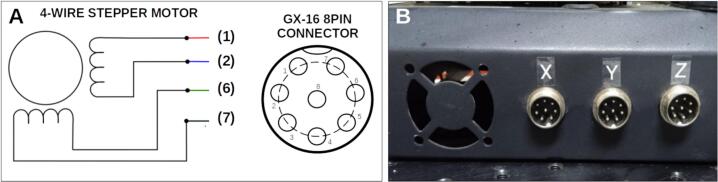
Wiring arrangement of the stepper motors and endstop sensors after modification. (A) Schematic diagram showing the connection of stepper motors to GX-16 connectors. (B) Photograph of the final installation, showing connector placement on the back of the 3D printer.

To connect the stepper motors to the main control unit, start by drilling the rear section of the 3D printer frame to install three GX-16 male connectors. Next, rewire the X, Y, and Z stepper motors and connect them to the corresponding connectors, following the wiring diagram shown in Fig. 21.A.

### 5.4 Main control unit build

**Fig. 22:**
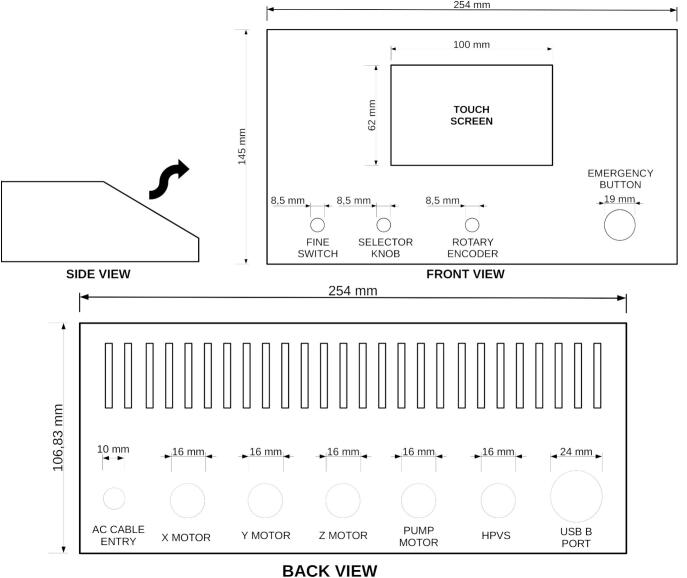
Drawing of the main control cabinet: side, from and back view.

To assemble the main control unit, use the circuit diagram in Fig. 8 as a reference. An old laboratory power supply case with a sloped design was repurposed to house the components, though any suitable enclosure listed in the BOM may be used. The front panel must be drilled according to the layout shown in the front view of Fig. 22, which details the positions and diameters for mounting the touchscreen (secured with a 3D-printed screen frame), rotary encoder, selector knob, HMI switch, and emergency button.

**Fig. 23:**
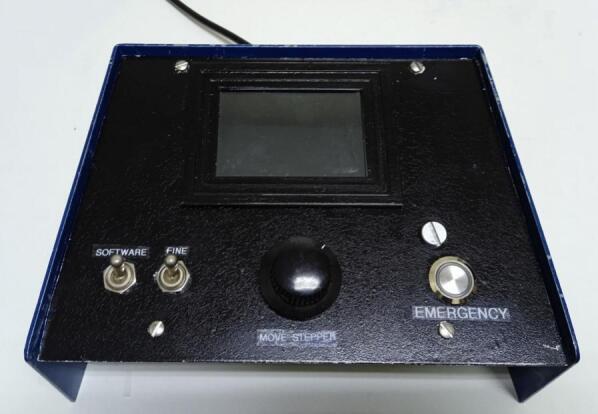
Front view of the main control unit.

The required cutouts for the GX16 ports, AC power entry, HPVS, and USB connections are shown in the back view of Fig. 22. While the exact placement can be adapted to the chosen enclosure, the hole sizes should remain as specified. Fig. 23 illustrates the fully assembled front panel of the control unit as a reference for the final build.

### 5.5 Acrylic enclosure

**Fig. 24:**
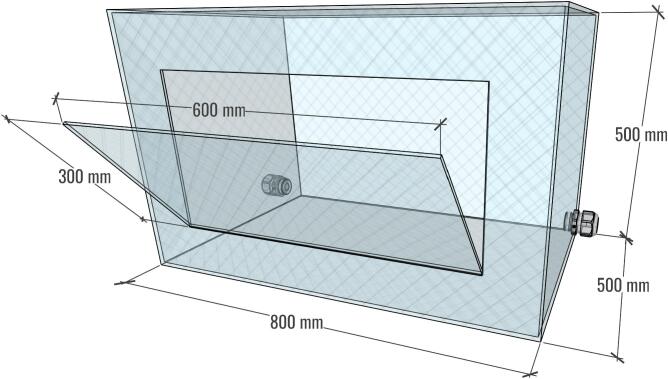
Acrylic enclosure diagram; lid and cable glands may be positioned as needed by the user.

**Fig. 25:**
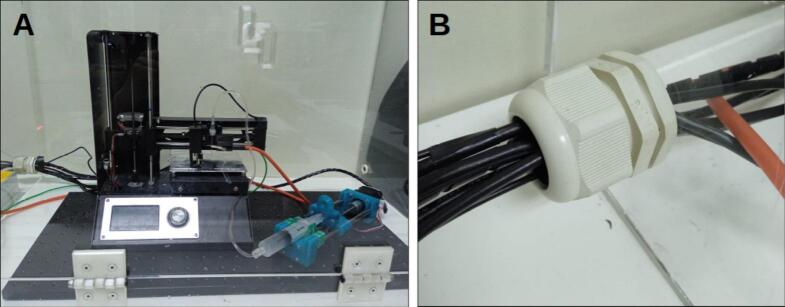
Acrylic enclosure. A) Mobile collector inside the acrylic enclosure. B) Cable gland to manage the device cables.

For the acrylic enclosure, the dimensions of the box are provided in Fig. 24. To manage the cables for the XYZ mobile collector and the HVPS, two holes were drilled on the side of the enclosure. Two cable glands with a 20 mm inner diameter each were then installed, as seems in Fig. 25. These cable glands can be positioned according to the layout of your equipment, ensuring efficient cable management.

## 6 Operation instructions

These steps outline the initial setup for an experimental session:

### 6.1 XYZ mobile collector setup

**Fig. 26:**
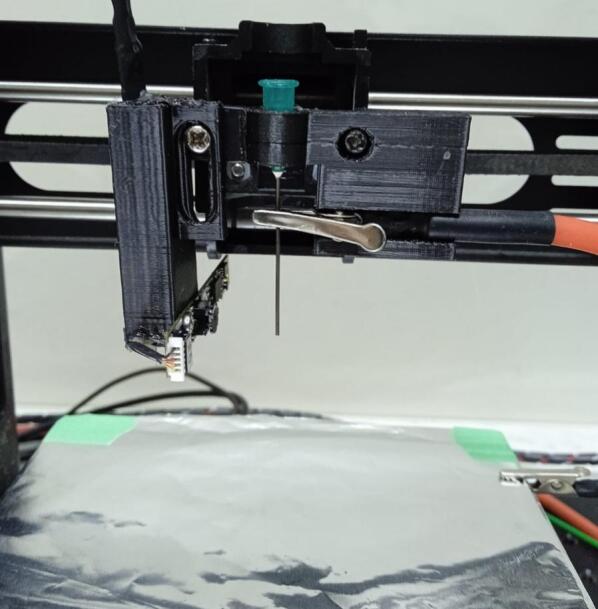
XYZ Collector Setup Preparation: Aluminium foil is placed on the base stage, and the needle was adjusted to align with the camera lens.

1. Start by cutting and placing aluminium foil on the XYZ mobile collector base. Connect the ground cable to it.
2. Turn on the main control unit without the needle placed.
3. Utilise the control software to move along the XY plane until the endpoint of the needle support is centred on the base. Then, set the Z height to 0.
4. Measure the distance between the stage and the bottom part of the needle support to determine the needle's length.
5. Trim the needle to match the height of the needle support. Adjust the camera support's height to align the needle tip accurately. Fine-tune the focus on the needle tip by adjusting the lens. Use the control software's camera streaming to monitor the process.
6. Secure the HVPS electrode to the needle. It should seem as shown in Fig. 26.

### 6.2 Syringe pump setup

1. For efficiency and speed, manually fill the syringe with the polymeric solution (Fig. 27.A); alternatively, use the bidirectional capability of the syringe pump to automate this process.

**Fig. 27:**
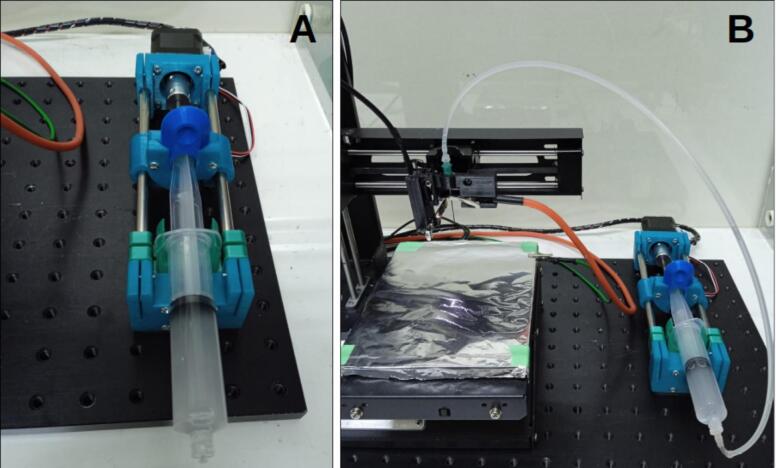
Syringe pump system configuration. (A) Syringe loaded with the polymeric solution. (B) Syringe connected to the needle via flexible tubing.
2. Use the manual control of the syringe pump to position its carriage and fit the syringe securely, using the plunger tab for attachment.
3. Connect the tubing to the end of the syringe and the needle at the other end.(Fig. 27.B).
4. Use the manual control without fine adjustments to start pumping the solution through the tubing. Alternatively, initiate a pumping protocol using either the software control or the touchscreen interface.
5. Activate the fine control (switch “Fine” on the main control unit) when the polymer solution reaches the top of the needle, and continue injecting until a small drop forms at the needle tip.
6. Expect some leakage depending on the solution's viscosity. Consider placing a paper tissue beneath the needle to prevent any drops from reaching the base.

### 6.3 Experiment operation

1. Place a glass slide at the centre of the mobile collector.
2. Use software stage control to locate the slide corners for reference, restricting your working area.
3. Activate the “Learn” option and map a route for the needle by either clicking on the grid or entering coordinates manually.
4. Turn off “Learn” mode and move the needle to a stage corner.
5. Set and start the pumping protocol of the syringe pump at the desired flow rate.
6. Switch on the HVPS and set the desired voltage. Gradually increase the voltage to obtain the Taylor cone desired shape. Utilise camera streaming to visualise the Taylor cone while being formed.
7. Start the listed protocol after the desired shape is achieved.
8. After completing the protocol, turn off both the HVPS and the syringe pump.

### 6.4 Safety warning

1. Before manipulating the acrylic encore interiors, ensure the HVPS is turned off using both the physical power switch and the software on/off button.
2. After completing the protocol route, always turn off the HVPS. Do not open the acrylic enclosure lid while the operation is ongoing.
3. Ensure that you set the needle height correctly. You may run a trial experiment without the pumping protocol to check and fix the route.

## 7 Validation and characterization

### 7.1 Cone Taylor and jet formation

To investigate the influence of the applied voltage and the needle-to-collector distance on the formation of the Taylor cone and jet behavior, experiments were performed using a polymeric solution of polyethylene oxide (PEO) at an 8 % concentration in distilled water. The feed rate was kept constant at 33  µL/min, empirically determined as the minimum value that ensured a stable droplet at the needle tip under all tested conditions, thereby preserving the capillary pressure required to balance the electrostatic forces and facilitating the generation of a thin, continuous jet. This strategy effectively minimized variability arising from intermittent dripping or meniscus instabilities, ensuring that the observed effects could be directly attributed to variations in voltage and distance.

**Fig. 28:**
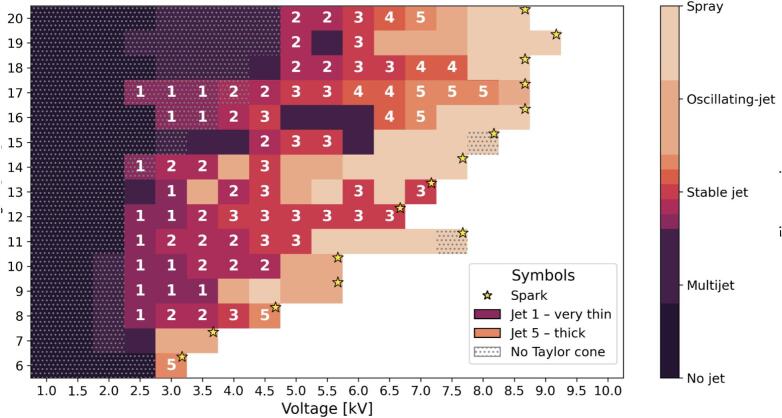
Phase map showing the influence of applied voltage and needle-to-collector distance on Taylor cone and jet formation at a fixed feed rate of 33  µL/min. Each region indicates a distinct flow regime: no jet, multijet, stable jet (graded from 1 to 5 from very thin to thick), oscillating jet, spray, or electrical discharge (spark). The numbers within the stable jet regions denote the relative jet thickness, while star symbols indicate occurrences of spark events.

Under these controlled parameters, the applied voltage and the needle height were systematically varied to construct a comprehensive phase map (Fig. 28). This map illustrates transitions among distinct flow regimes, including: absence of a jet (where the electric field was insufficient to deform the droplet), multijet formations (multiple emission sites due to excessive surface charge), stable jets (classified on a scale from 1 to 5, indicating increasing jet thickness from very thin to thick), oscillating jets (resulting from lateral electrostatic instabilities), spray, and electrical discharges (sparks). This detailed classification of stable jets provides further insights into how process parameters modulate jet morphology, which is directly linked to the resulting fiber dimensions and alignment.

**Fig. 29:**
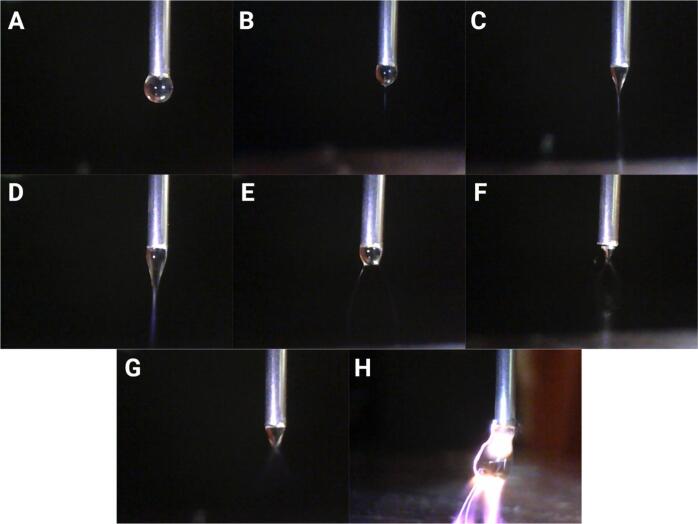
Representative images of Taylor cone and jet regimes at 33  µL/min: (A) no Taylor cone (2.5  kV, 11  mm height); (B) stable Taylor cone with jet 1 – very thin (2.5  kV, 11  mm height); (C) stable Taylor cone with jet 3 (4.5  kV, 11  mm height); (D) stable Taylor cone with jet 5 – thick (7  kV, 17  mm height); (E) multijet from Taylor cone (3  kV, 10  mm height); (F) oscillating Taylor cone jet (5  kV, 9  mm height); (G) spray from Taylor cone (6.5  kV, 11  mm height); (H) spark disrupting Taylor cone (3  kV, 6  mm height).

Representative images of each regime, as captured during the experiments, are presented in Fig. 29. These illustrate the characteristic droplet shapes, Taylor cone geometries, jet structures, multijet scenarios, oscillations, spray patterns, and spark events observed under the different operational windows identified in the phase map.

In the first regime(Fig. 29A), corresponding to low applied voltages or large needle-to-collector distances, the electric field generated between the needle and the collector was insufficient to overcome the surface tension of the liquid droplet at the needle tip. As a result, the droplet retained its nearly spherical shape without elongating into a Taylor cone, and consequently, no jet was initiated. In this regime, the electrostatic forces were simply too weak to deform the droplet interface against the restoring capillary pressure [31].

By incrementally raising the applied voltage or shortening the nozzle-to-collector gap—thereby intensifying the electric field—the electrostatic stress eventually equals the capillary force that holds the droplet intact. At this threshold the droplet elongates into a stable Taylor cone that emits a continuous, straight jet (Fig. 29C-E). This cone-jet state delineates the optimal operating window for near-field electrospinning: with gaps of a few millimetres, the jet remains unbent, allowing the direct writing of fibres whose diameters and alignment are tightly controlled. Operating just above the threshold maximises pattern fidelity and reproducibility while suppressing secondary oscillations that would otherwise degrade fibre alignment quality [32].

Pushing the electric field beyond the optimal window gives rise to two distinct, charge-driven instabilities. First, the droplet may enter a multijet regime: as surface charge increases, several filaments erupt from the Taylor cone; most die out rapidly while one survives and stabilises, a transient behaviour often observed just before a steady single-jet is established. If the field (or the nozzle diameter) is raised further, charge accumulation makes multiple emission sites energetically favourable and several jets persist simultaneously, fragmenting the current and breaking the axisymmetry of the cone (Fig. 29B). This multijet state can therefore appear either as a brief precursor to the stable jet or as a sustained high-voltage instability, corroborating reports that excessive charge concentration, rather than flow-rate limitations, triggers the phenomenon [33].

Beyond this threshold, the bending-whipping instability dominates. Here the lateral Coulombic repulsion between like charges on the jet surface exceeds the axial viscoelastic tension, causing small off-axis perturbations to grow into vigorous whipping motions (Fig. 29F). The jet oscillates, forming expanding coils that stretch and thin the filament while disrupting its alignment on the collector.[34]. These “whipping motions” explicitly deal with the imbalance between repulsive electrostatic forces and the jet’s viscoelastic resistance, emphasising that higher viscosities can damp the oscillations without eliminating the underlying electrostatic driving force. This mechanistic view is consistent with experimental trends showing increased whipping amplitude with voltage and spinneret–collector distance, and its mitigation by more viscous or entangled solutions [35].

Pushing the system even further—by applying excessive voltages or reducing the needle-to-collector gap too much—caused the charged jet to exceed its Rayleigh stability limit, breaking up into microdroplets typical of electrospray behavior [36]. In some cases, the electric field also surpassed the dielectric breakdown of air, resulting in intermittent sparks across the gap that completely destabilized the process.

### 7.2 Fiber scaffold testing

**Fig. 30:**
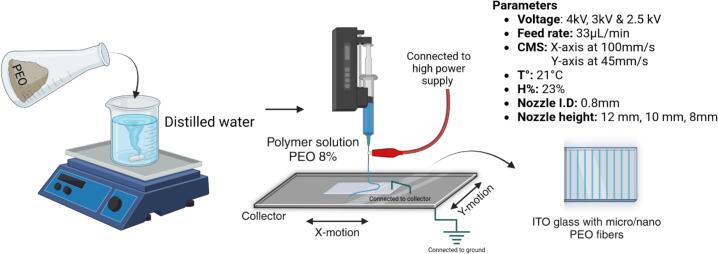
Experimental setup for electrospinning process using a PEO solution, deposited onto an ITO glass substrate with varying applied voltages (4 kV, 3 kV and 2.5 kV) and needle-to-collector distances (12 mm, 10 mm, 8 mm).

To evaluate the performance of the device, electrospun fiber scaffolds were fabricated using the same polymeric solution of PEO at an 8 % concentration dissolved in distilled water. A 20 mL BD Plastipak™ syringe (polypropylene, Luer Lock, internal diameter 19.13 mm) was used to deliver the solution onto a square ITO glass substrate, which was connected to the grounded collector via a thin nickel wire. The fabrication process, illustrated in Fig. 30 and conducted under the experimental conditions specified in Table 8, explored the effects of varying applied voltages (2.5, 3, and 4 kV) and different needle-to-collector distances (8, 10, and 12 mm) on fiber morphology and alignment.

**Table 8:** Specifications of the parameters used to produce the sample 1 using near-field electrospinning.

**Sample 1 Parameters**	**Value**
Polymer solution	PEO/water 8 %
Deposition substrate	ITO glass 15 mm x 15 mm
Needle height	12 mm, 10 mm, and 8 mm
Voltages	4 kV, 3 kV, and 2.5 kV
Fiber spacing	0.5 mm
X-axis speed	100 mm/s
Y-axis speed	45 mm/s
Syringe flow rate	33 µL/min
Ambient temperature	21 °C
Relative ambient humidity	53 %

A flow rate of 33 µL/min was empirically determined as the minimum required — considering the viscosity of the polymer solution and the specific height and voltage settings — to sustain a small droplet at the needle tip, which provided the base for forming a very thin and stable jet. Throughout the experiments, this flow consistently maintained the droplet, ensuring steady jet formation.

**Fig. 31:**
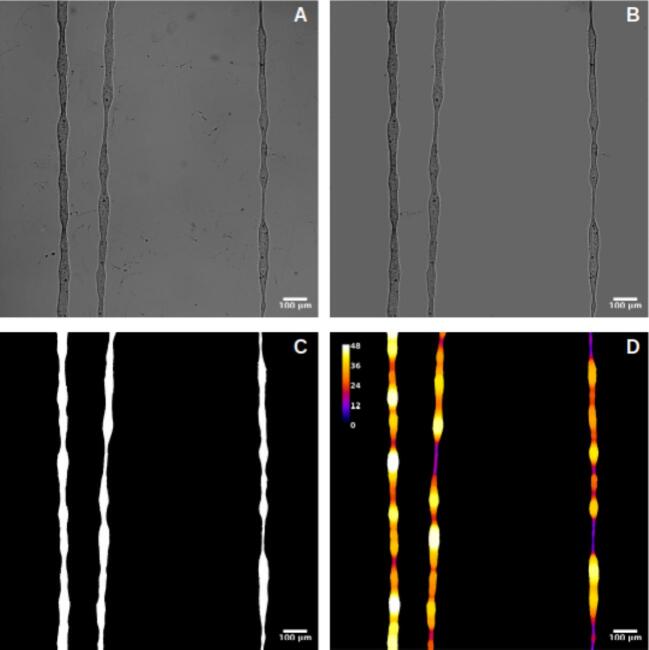
Optical microscopy images and analysis of deposited electrospun fibers at 4 kV. From left to right: 8 mm, 10 mm, and 12 mm needle height. (A) Raw image. (B) Background-corrected image. (C) Binary mask of the fibers. (D) Thickness analysis map. Thermal scale is on micrometers.

**Fig. 32:**
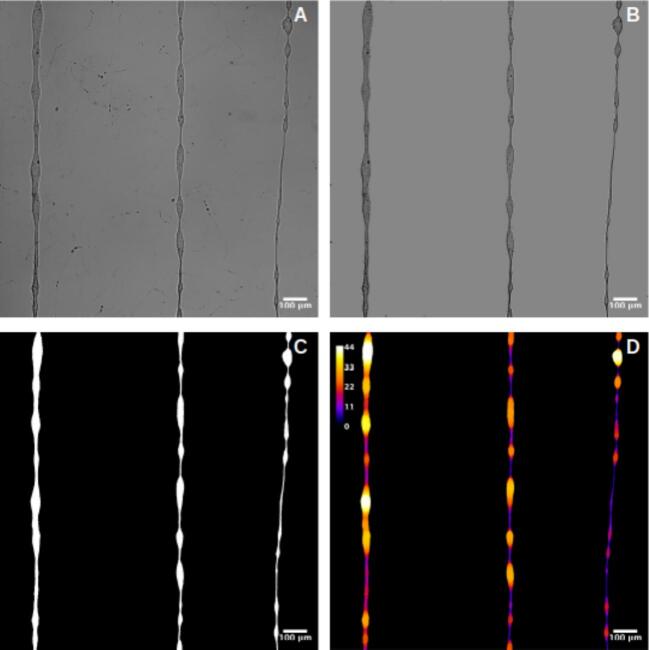
Optical microscopy images and analysis of deposited electrospun fibers at 3 kV. From left to right: 8 mm, 10 mm, and 12 mm needle height. (A) Raw image. (B) Background-free image. (C) Binary mask of the fibers. (D) Thickness analysis map. Thermal scale is on micrometers.

**Fig. 33:**
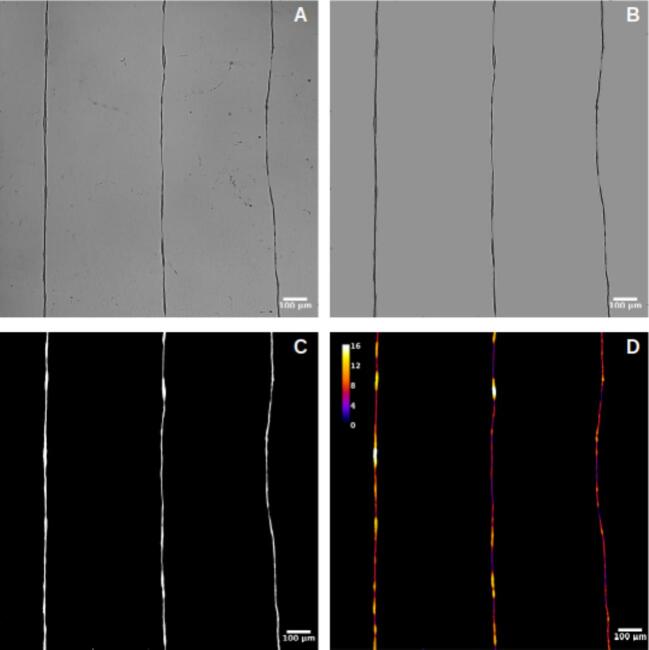
Optical microscopy images and analysis of deposited electrospun fibers at 2.5 kV. From left to right: 8 mm, 10 mm, and 12 mm needle height. (A) Raw image. (B) Background-free image. (C) Binary mask of the fibers. (D) Thickness analysis map. Thermal scale is on micrometers.

For the thickness analysis, raw images were imported into FIJI, where the background was removed to isolate the fibers, as shown in Fig. 31b, Fig. 32b, and Fig. 33b. A mask was then created by adjusting the contrast and applying a threshold to generate a binary image, as depicted in Fig. 31, Fig. 32, Fig. 33. The Local Thickness plugin was applied to calculate the diameter of the largest circle that could fit inside each fiber at a given point. This algorithm computes a Euclidean distance map of the foreground pixels, processes it into a distance ridge, and produces a local thickness map [37]. The resulting heat map displays warmer colors for thicker areas and cooler colors for thinner areas, allowing for the assessment of fiber diameter distributions.

**Fig. 34:**
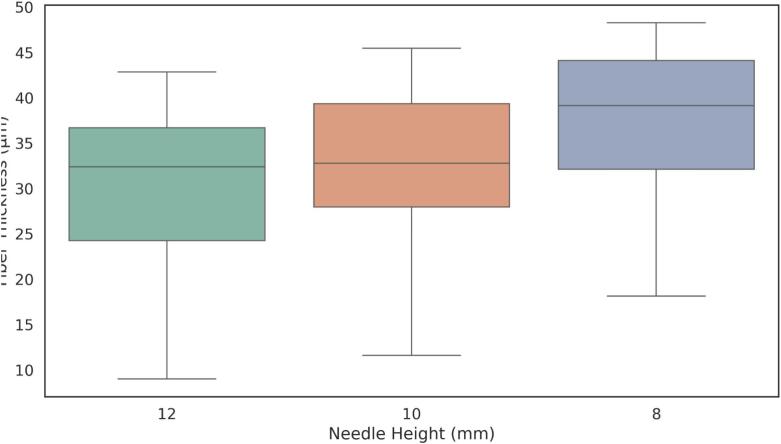
Fiber thickness distribution obtained in sample 1 at a voltage of 4 kV, evaluated at different needle heights: 12 mm, 10 mm, and 8 mm.

**Fig. 35:**
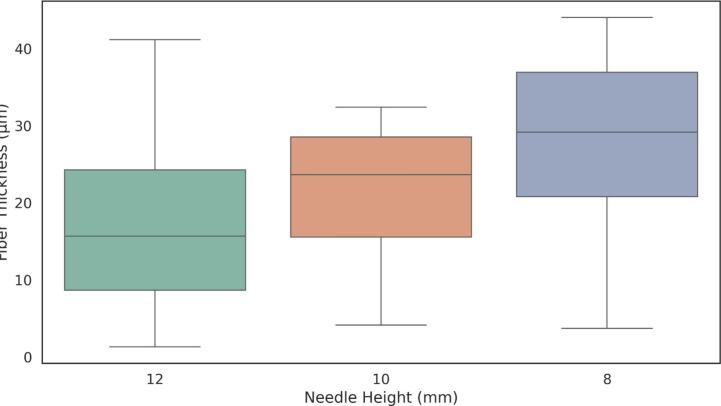
Fiber thickness distribution obtained in sample 2 at a voltage of 3 kV, evaluated at different needle heights: 12 mm, 10 mm, and 8 mm.

**Fig. 36:**
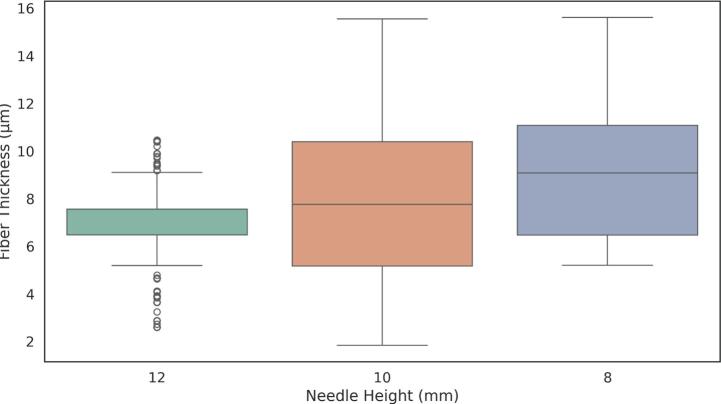
Fiber thickness distribution obtained in sample 3 at a voltage of 2.5 kV, evaluated at different needle heights: 12 mm, 10 mm, and 8 mm.

Histogram data of pixel intensity values for each fiber were exported, and box plots were created, as shown in Fig. 34, Fig. 35, Fig. 36. Table 8 summarizes the statistical results, including the minimum, mean, and coefficient of variation values.

**Fig. 37:**
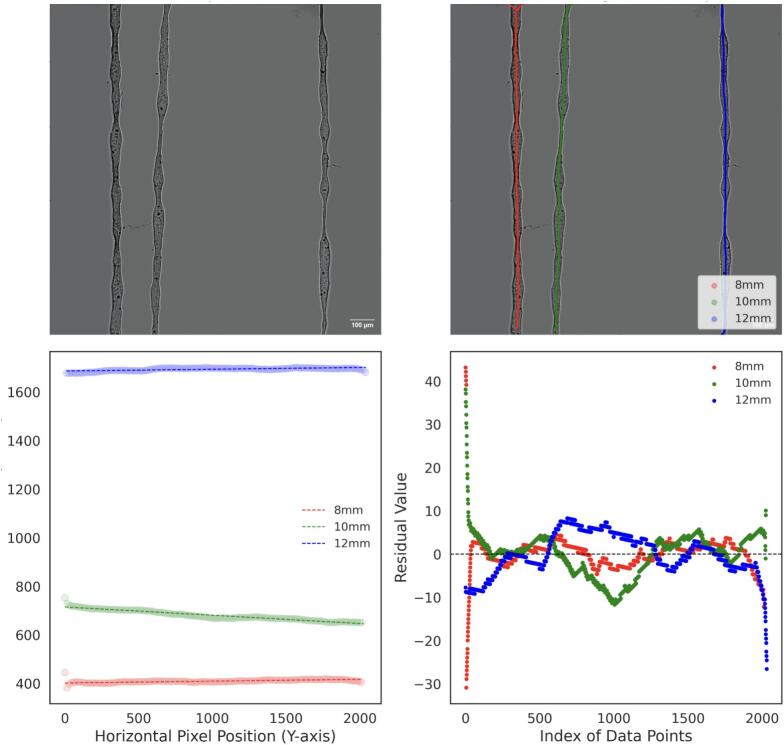
Analysis of electrospun fiber straightness at 4 kV. (A) Background-free image of electrospun fibers. (B) Skeletonized and segmented image, showing individual fiber paths (C) Linear fit applied to each segmented fiber path. (D) Residual analysis for deviations from the linear fit.

**Fig. 38:**
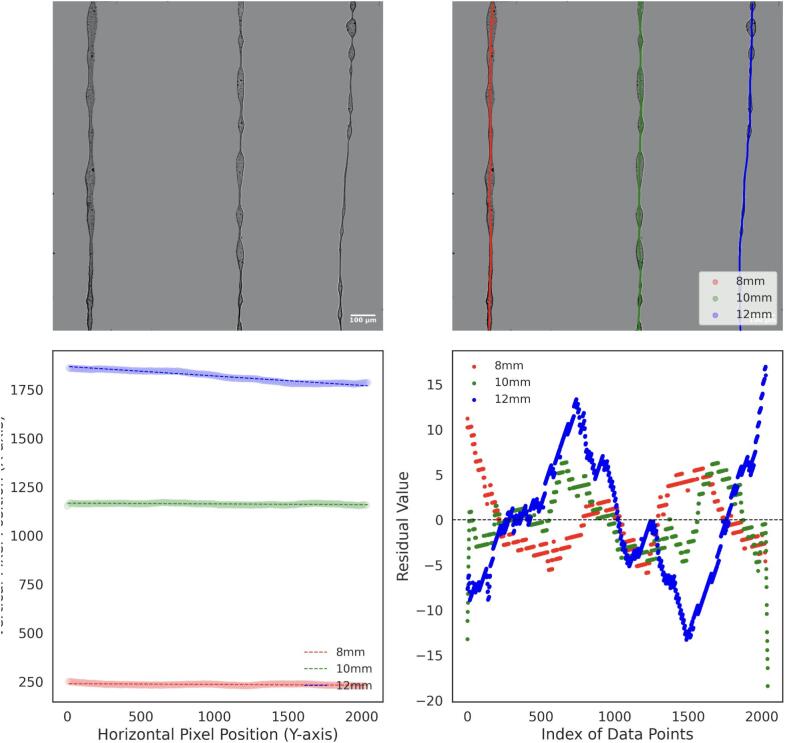
Analysis of electrospun fiber straightness at 3 kV. (A) Background-free image of electrospun fibers. (B) Skeletonized and segmented image, showing individual fiber paths (C) Linear fit applied to each segmented fiber path. (D) Residual analysis for deviations from the linear fit.

**Fig. 39:**
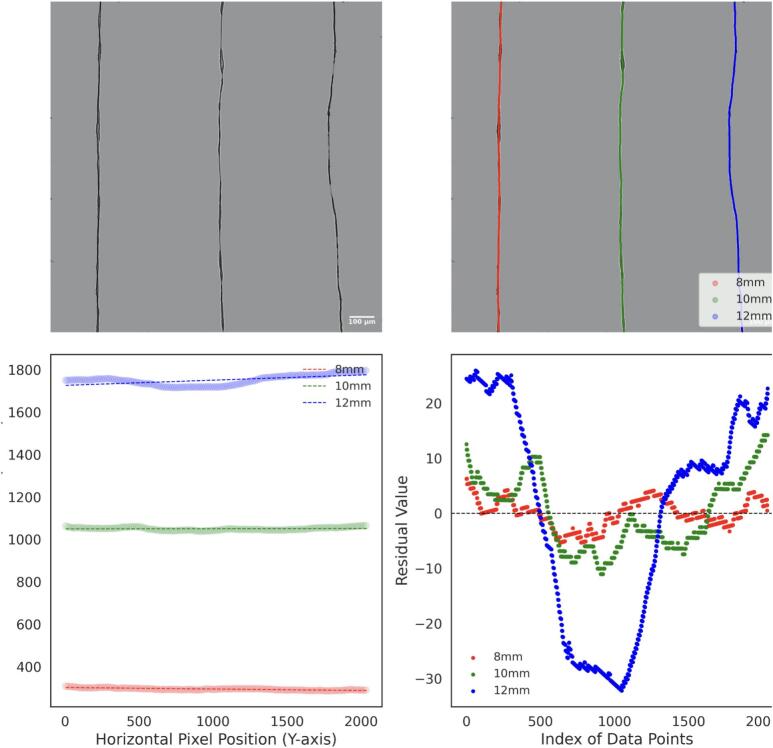
Analysis of electrospun fiber straightness at 2.5 kV. (A) Background-free image of electrospun fibers. (B) Skeletonized and segmented image, showing individual fiber paths (C) Linear fit applied to each segmented fiber path. (D) Residual analysis for deviations from the linear fit.

To analyze the straightness of the electrospun fibers, background-free images were skeletonized using Fiji and processed with a Python algorithm. The algorithm employs DBSCAN clustering to segment the skeletonized fibers into distinct lines based on the spatial density of pixels, allowing for the identification and separation of individual fiber paths. These paths were then labeled by position (8 mm, 10 mm, and 12 mm), as shown in Fig. 37b, Fig. 38b, and Fig. 39b, with each detected line ordered from left to right for labelling.

A linear regression was performed for each separated fiber path. The horizontal (x) and vertical (y) coordinates of each line were extracted, and a linear model was fitted to the data points. The electrospun fiber paths and their corresponding fitted lines are shown in Fig. 37, Fig. 38, Fig. 39.

A residual analysis was then conducted by calculating the difference between the actual data points and the predicted values from the linear fit. These residuals, shown in Fig. 37, Fig. 38, Fig. 39, were used to evaluate the uniformity of fiber straightness. Higher residuals indicate greater deviations from the linear fit, suggesting misalignment.

Finally, the results were summarized in Table 10, which includes the regression parameters and residuals, ranking the standard deviation and highlighting the minimum value obtained.

**Table 9:** Summary of mean fiber thickness, standard deviation, and coefficient of variation (CV) for electrospun fibers under different voltages and needle heights.

**Voltage (kV)**	**Needle Height (mm)**	**Mean Thickness (µm)**	**Std Dev (µm)**	**Coeff. of Variation (%)**
4	12	29.823	9.099	30.512
4	10	33.228	8.246	24.817
4	8	37.69	7.56	20.057
3	12	18.014	11.058	61.384
3	10	21.734	7.917	36.428
3	8	28.717	9.331	32.494
2.5	12	6.568	1.379	20.995
2.5	10	8.084	3.154	39.016
2.5	8	9.335	2.666	28.554

**Table 10:** Summary of linear fit and residual analysis for electrospun fibers under different voltages and needle heights.

**Voltage**	**Needle Height**	**Slope**	**Intercept**	**Mean Residual**	**Residuals Std Dev**	**Residuals Max**	**Residuals Min**	**Ranked Std Dev**
2.5 kV	8 mm	−0.007	301.771	−1.622E-14	**2.603**	6.292	−5.313	1
2.5 kV	10 mm	0.001	1049.426	−8.093E-14	6.436	14.218	−11.051	7
2.5 kV	12 mm	0.024	1725.413	−8.492E-15	19.607	25.947	–32.216	9
3 kV	8 mm	−0.004	238.832	−1.685E-14	3.495	11.213	−5.875	3
3 kV	10 mm	−0.004	1166.216	1.249E-13	3.184	6.37	−18.403	2
3 kV	12 mm	−0.049	1868.364	9.512E-14	6.643	16.96	−13.296	8
4 kV	8 mm	0.008	401.812	−2.487E-15	3.807	43.188	−30.85	4
4 kV	10 mm	−0.034	714.909	−2.285E-14	5.031	38.125	−11.675	6
4 kV	12 mm	0.007	1686.621	−7.266E-14	4.7	8.29	−26.544	5

Overall, the results indicate that lower applied voltages produce thinner fibers, while increasing the voltage to 4  kV leads to thicker fibers. Additionally, lower needle heights, particularly at 8  mm, resulted in greater variability in fiber alignment, suggesting that the electrospinning process was more stable under these conditions.

The optimal parameters for generating well-aligned and uniform fibers were found at a needle height of 8  mm: 4  kV produced thicker fibers, whereas 2.5  kV yielded thinner fibers, both achieving the lowest coefficient of variation in diameter (15 %) and minimal deviations from linear alignment.

The operational window that enables the formation of either thin or thick fibers is clearly represented in the Taylor-cone phase diagram (Fig. 28). At 2.5  kV and 8  mm, the system operates in the “Jet 1” sub-region, just above the cone-jet threshold. Here, the jet emerges with a minimal initial radius; surface tension dominates over axial charge, maintaining laminar flow. This balance allows the filament to solidify before lateral instabilities develop, resulting in thin, straight, and highly uniform fibers with a coefficient of variation around 15 % (Table 9)—ideal for biosensing applications requiring high surface-to-volume ratios.

Raising the electric field to 4  kV at the same 8  mm gap shifts the process into the “Jet 3” region. The cone sharpens, and the increased charge density thickens the jet before solidification. However, the very short flight time characteristic of NFES (≈ 100  µs) limits the growth of disturbances, preventing the whipping seen in far-field electrospinning. The fibers produced are three to four times thicker yet maintain residual misalignments below 5  µm, making them well suited for mechanically robust scaffolds.

A field-to-gap ratio of roughly 0.3–0.5  kV mm^−1^ thus defines two stable NFES sub-windows—“fine-jet” (Jet 1) and “thick-jet” (Jet 3)—allowing precise control over fiber diameter without compromising alignment, provided the flow rate remains at the droplet-threshold value of 33  µL min^−1^ to sustain capillary balance.

Further exploration of parameters such as polymer concentration, ambient temperature, humidity, and different polymers could enable the fabrication of even thinner, better-aligned fibers, broadening the capabilities of this system.

**Fig. 40:**
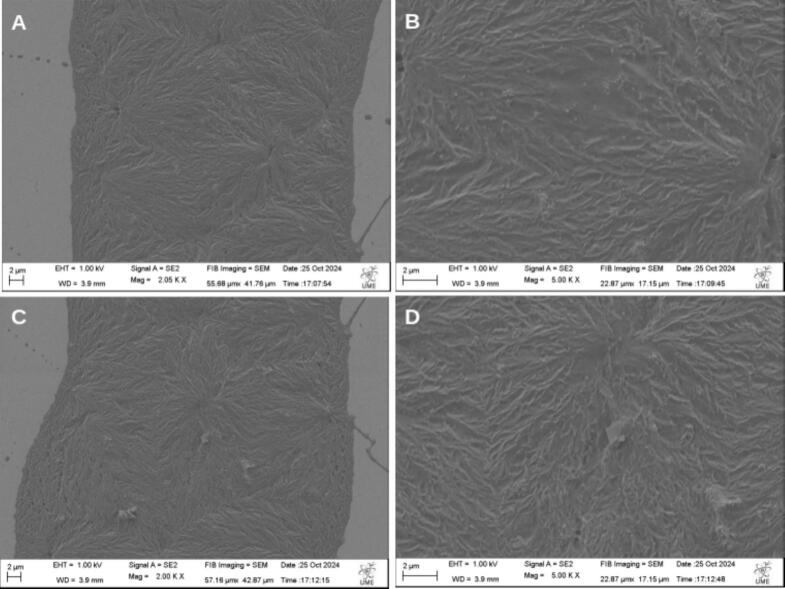
Scanning electron microscopy (SEM) images of the top surface of fibers produced by the NFES. (A) Overview of Section 1 at lower magnification (2.05 K X), obtained at 1 kV and 3.9 mm working distance. (B) Close-up view (5.00 K X) of Section 1, highlighting fiber morphology and surface details, obtained at 1 kV and 3.9 mm working distance. (C) Overview of Section 2 at lower magnification (2.00 K X), obtained at 1 kV and 3.9 mm working distance. (D) Close-up view (5.00 K X) of Section 2, obtained at 1 kV and 3.9 mm working distance.

**Fig. 41:**
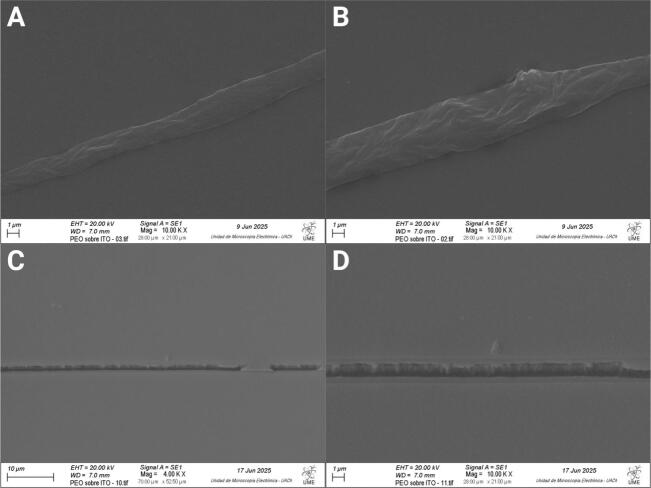
SEM images of the top surface of thinner fibers produced by the NFES. (A) Overview of Section 1 at 4.00 K X magnification, obtained at 20 kV, and 7.0 mm working distance (B) Close-up view (10.00 K X) of Section 1, highlighting fiber morphology and surface details, obtained at 20.00 kV and 7.0 mm working distance (C) Overview of Section 2 at 4.00 K X magnification, obtained at 20.00 kV and 7.0 mm working distance.(D) Close-up view (10.00 K X) of Section 2, obtained at 20.00 kV and 7.0 mm working distance.

Additionally, scanning electron microscopy (SEM) confirmed that all fibers, regardless of voltage or needle height, exhibited consistent morphology, reinforcing the reliability and reproducibility of the NFES process. The SEM images illustrating these results are shown in Fig. 40 and Fig. 41.

### 7.3 Functionalization of fibers

**Fig. 42:**
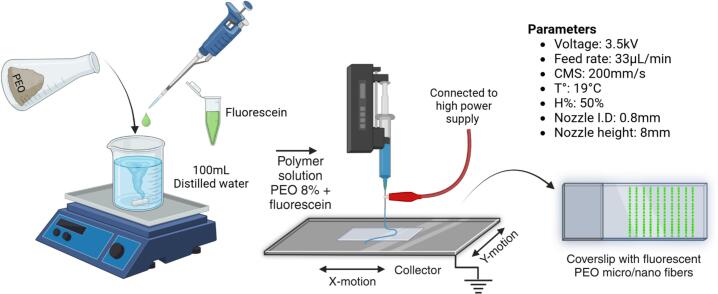
Experimental setup for achieving a fluorescent PEO fibers scaffold.

The porous nature of the fibers observed at SEM motivates us to devise a proof of concept for fiber functionalization. Fluorescent fibers were successfully obtained by simply incorporating the organic dye fluorescein into the polymer solution. Fluorescein can be excited at a peak wavelength of 491 nm, emitting a bright green fluorescence with a peak wavelength at 516 nm. This experiment demonstrates the feasibility of biofunctionalizing the fibers. The incorporation of fluorescein not only highlights the potential for integrating small organic molecules into the fibers but also lays the groundwork for their application as biosensors. Fig. 42 illustrates the experimental setup used to validate this proof of concept.

**Fig. 43:**
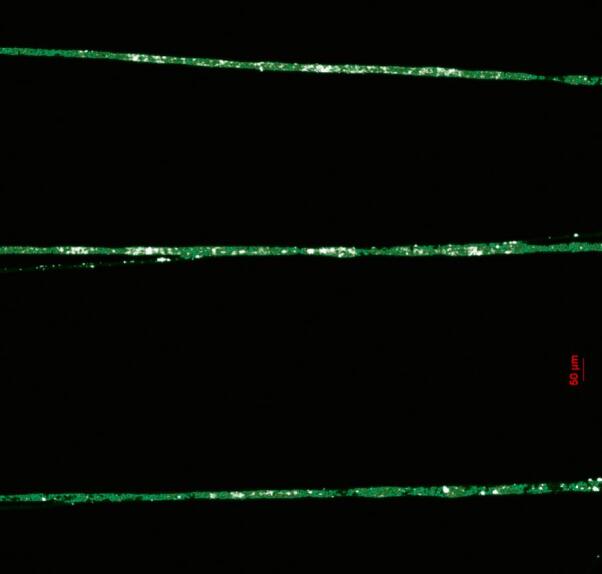
Fluorescent electrospun fibers produced by the NFES system, showing three well-aligned fibers emitting green fluorescence under excitation at 491 nm. (For interpretation of the references to colour in this figure legend, the reader is referred to the web version of this article.)

The results are shown in Fig. 43, displaying three well-aligned fibers emitting green fluorescence suggesting that the optical properties of the dye were preserved even after the experimental procedure that included a high-voltage arc. The high degree of alignment achieved in the fibers further demonstrates the capability of the NFES to produce uniform and precisely positioned fibers, which is critical for future biosensing applications.

## Ethics statements

The work did not involve the use of any human or animal subjects.

## CRediT authorship contribution statement

**Cristian Castillo-Velásquez:** Writing – original draft, Validation, Software, Methodology, Investigation, Formal analysis, Conceptualization. **Carlos Fuhrhop:** Writing – review & editing, Supervision, Resources, Project administration, Investigation, Funding acquisition, Conceptualization. **Mario E. Flores:** Writing – review & editing, Resources. **Sebastian Brauchi:** Writing – review & editing, Supervision, Resources, Funding acquisition, Data curation, Conceptualization.

## Declaration of competing interest

The authors declare that they have no known competing financial interests or personal relationships that could have appeared to influence the work reported in this paper.
